# Ageism toward older adults during COVID-19: associations with emotional coping and intergenerational contact

**DOI:** 10.3389/fpsyg.2026.1949869

**Published:** 2026-10-15

**Authors:** Theresa L. Scott, Melissa Kugelman, Amanda Salmon

**Affiliations:** Faculty of Health, Medicine and Behavourial Sciences, School of Psychology, The University of Queensland, Brisbane, QLD, Australia

**Keywords:** aging expectations, ageism, benevolent, contact hypothesis, hostile, survey

## Abstract

**Introduction:**

The COVID-19 pandemic brought substantial social and emotional stressors, alongside widespread messaging that framed older adults as particularly vulnerable. It remains unclear whether these conditions were associated with hostile and benevolent ageist perspectives of older people. Thus, this study aimed to investigate the factors associated with ageism, perceived changes in emotional coping, and the attitudes and experiences of Australian residents toward older adults.

**Methods:**

Ageism, aging expectations, and contact quality with older adults in the context of COVID-19 were explored using an online, cross-sectional correlational study design, participants responded to both established and novel measures for these factors.

**Results:**

A total of 273 Australian residents completed the online study (aged 19–82 years, *M* = 40.73, *SD* = 14.74). There was no association between emotional coping and ageist beliefs. More positive aging expectations were related to lower hostile ageism, but not benevolent ageism. Positive contact with older adults was linked with lower hostile ageism and higher benevolent ageism, while negative contact showed the reverse pattern.

**Conclusion:**

The current study suggests that ageist messages portraying ‘vulnerability’ reflected commonly held attitudes toward older Australians during the pandemic and highlights the importance of considering both the quality and nature of intergenerational contact when developing ageism-reduction strategies. Potential explanations, limitations and directions for future research are discussed.

## 1 Introduction

The declaration of COVID-19 as a global pandemic in March 2020 significantly transformed public discussion of health, risk, and vulnerability (Savage et al., 2020). Within the Australian context, the pandemic had substantial social, emotional, and economic consequences, including widespread impacts on mental health (Biddle et al., 2020; Fisher et al., 2020). Older adults were central to this discourse, particularly in relation to their perceived vulnerability to severe illness and increased mortality.

In this context, health and mortality become increasingly salient, with potential implications for perceptions of aging and expected health in later life (Curșeu et al., 2021; Tyrer, 2020). In the United States, more negative expectations of aging were associated with more negative mood during the pandemic (Whatley et al., 2020). Further, these negative expectations of aging may be related to anxiety about aging and death, both of which have been identified as predictors of poorer mood (Curșeu et al., 2021; Lamont et al., 2015; Swift et al., 2017). Thus, these associations may have been intensified during the pandemic, when concerns about health, aging, and mortality were particularly prominent (Bellingtier and Neupert, 2018; Whatley et al., 2020).

Ageism was also identified as a predictor of increased aging anxiety (Curșeu et al., 2021). Researchers have highlighted a proliferation in ageist statements and beliefs about older adults during the COVID-19 pandemic across social media, news, and political discourse (Ayalon, 2020; Brooke and Jackson, 2020; Fraser et al., 2020; Martikainen and Sakki, 2021). In Australia, media reporting frequently emphasized the higher mortality rates for people over the age of 70, while public health advice encouraged older adults to physically distance from others (Australian Government Department of Health, 2021). While global epidemiological trends supported an association between older age and poorer outcomes, Vervaecke and Meisner (2020) emphasized that age alone was not a consistent predictor of COVID-19-related medical outcomes and cautioned that such rhetoric could reinforce harmful stereotypes. For example, portraying older adults as more susceptible to COVID-19 may have bolstered ageist stereotypes of older adults as “vulnerable,” strengthened internalized ageism, and facilitated paternalistic protective behaviors, with potential negative consequences for older people’s health and wellbeing (Ayalon, 2020; Meisner, 2020; Pachana et al., 2020; Swift et al., 2017).

Media coverage commonly reflected two polarized perspectives on COVID-19, each with significant implications for older adults (Martikainen and Sakki, 2021; Meisner, 2020). The first emphasized the need to follow preventative health measures to ‘protect’ older adults and ‘keep the vulnerable safe’, a framing that may have been perceived as patronizing and reinforced benevolent ageist stereotypes (Fraser et al., 2020; Vervaecke and Meisner, 2020). Conversely, hostile ageism was evident in arguments that society should learn to live with the virus, regardless of the resulting loss of lives (Brooke and Jackson, 2020; Pachana et al., 2020). This perspective often reflected the sentiment that the lives of older adults were less valuable, and that they represented a dispensable group (Meisner, 2020).

Research conducted during and following the COVID-19 pandemic has examined its impacts on older adults and attitudes toward aging (Brinkhof et al., 2023; Chasteen et al., 2023; Fraser et al., 2020; Meisner, 2020). Specifically, studies have investigated ageism, expectations regarding aging, and the experiences of older adults during the pandemic (Heid et al., 2021; Tse et al., 2021; Van Rheenen et al., 2020; Visintin, 2021; Whatley et al., 2020). However, less is known about how individuals’ experiences of pandemic-related stress were associated with ageist attitudes and expectations regarding aging within the Australian context. The current study therefore sought to examine the relationships between perceived changes in emotional coping, hostile and benevolent ageism, expectations regarding aging, and contact with older adults among Australian residents. Australia provided a particularly relevant context for examining these relationships because public health messaging during the pandemic frequently emphasized older adults as a distinct high-risk group, while the social and policy response also placed considerable emphasis on protecting older people.

### 1.1 Emotional toll

The COVID-19 pandemic had substantial effects on the Australian economy, communities, and individuals (Biddle et al., 2020; Kent et al., 2020; Van Rheenen et al., 2020). Research documented the effects of social restrictions and unemployment on mental health across a range of populations and circumstances (Churchill, 2021; Javed et al., 2020; Jones et al., 2020; McBride et al., 2020; Phillipou et al., 2020). In general, higher rates of mild to moderate symptoms of depression and anxiety were identified in a sample of Australian residents during April to May of 2020, relative to pre-pandemic estimates (Fisher et al., 2020). In the current study, emotional coping refers specifically to participants’ perceived ability to cope emotionally with the impacts of the pandemic. It was intended to capture perceived change in coping rather than symptoms of depression, anxiety, psychological distress, or broader psychological wellbeing.

### 1.2 Ageism

Beyond the direct emotional and financial toll of the pandemic, ageist messages became widespread across news and social media (Cohn-Schwartz and Ayalon, 2020; Swift and Chasteen, 2021). Ageism is most often defined as a set of attitudes, stereotypes, and/or discrimination toward an individual based on their chronological age, usually with respect to the consequences for older people (Barnett and Adams, 2018; Chonody, 2016; Meisner, 2020; Swift et al., 2017). ‘Older adults’ is commonly defined as those aged 65 years or older; however definitions of this age group vary across contexts and individual perceptions (Swift and Chasteen, 2021). Widely embedded portrayals of older adults as frail, dependent, and incompetent have been shown to have notable consequences for health and wellbeing, including negative impacts on mental health, physical functioning, and life expectancy (Kannoth, 2018; Officer et al., 2020). The Risks of Ageism Model (RAM) describes three key pathways by which ageism impacts older people: stereotype embodiment, stereotype threat, and age-based discrimination (Swift et al., 2017). While stereotype threat may be triggered in specific situations where an individual feels at risk of confirming a negative stereotype, stereotype embodiment is thought to operate more consistently and implicitly over time (Fawsitt and Setti, 2017; Swift et al., 2017). Moreover, discrimination based on age has been identified in a range of contexts, including healthcare and employment settings (Brooke and Jackson, 2020; Monahan et al., 2020; Swift et al., 2017).

#### 1.2.1 Internalized ageism

Stereotype embodiment, and specifically internalized ageism, may have intensified during the COVID-19 pandemic and has been proposed as one explanation for some of its disproportionate effects on older adults (Monahan et al., 2020; Steward, 2021; Swift and Chasteen, 2021). Messages that older adults were more susceptible to COVID-19 likely reinforced stereotypes of vulnerability, poor health, and helplessness, thus increasing the salience of these ideas (Steward, 2021). Research has found that people with more negative self-perceptions of aging engaged in fewer preventative health behaviors, and experienced shorter longevity, increased cardiovascular stress, and reduced physical functioning (Steward, 2021; Levy and Leifheit-Limson, 2009; Levy et al., 2014). Thus, homogenizing health messages about COVID-19 may have reinforced stereotypes, strengthened internalized ageism, and inadvertently discouraged preventative health behaviors (Steward, 2021).

#### 1.2.2 Hostile and benevolent ageism

Ageism research has identified two primary forms of ageist attitudes: hostile ageism and benevolent ageism. Hostile ageist beliefs are often explicit in nature and involve attitudes that older adults are cold, burdensome, and selfish (Graf and Knepple Carney, 2021). In contrast, benevolent ageism is more paternalistic and endorses stereotypes that older adults are incompetent, weak, or frail and require support or care (Cary et al., 2017; Vervaecke and Meisner, 2020). Cohn-Schwartz and Ayalon (2020) identified two common narratives about older adults in COVID-19-related media: the ‘vulnerability narrative’ and the ‘burden narrative’. The latter, reflecting hostile ageism, was evident in social media discourse that described COVID-19 as the ‘#BoomerRemover’, implying that the disproportionate mortality of older adults was acceptable, alongside portrayals of older adults as a drain on resources during the pandemic (Brooke and Jackson, 2020; Fraser et al., 2020; Meisner, 2020; Vervaecke and Meisner, 2020). The ‘vulnerability narrative’, reflecting benevolent ageism, framed older adults as dependent, helpless, and incapable, and was evident in messaging centered the need to ‘protect’ older adults (Ayalon, 2020; Brooke and Jackson, 2020; Fraser et al., 2020). This perspective portrayed older adults as broadly vulnerable to COVID-19 and contributed to the harmful homogenization of a diverse group of people (Ehni and Wahl, 2020; Fraser et al., 2020; Meisner, 2020). It also reflected the generalization that all older adults are equally susceptible to COVID-19, while implying that younger adults were less likely to contract the disease (Ayalon, 2020; Fraser et al., 2020; Vervaecke and Meisner, 2020). These messages may have further reinforced stereotypes of older adults as frail, vulnerable, and care-dependent (Ehni and Wahl, 2020; Meisner, 2020; Visintin, 2021).

The Stereotype Content Model (SCM) proposes two dimensions for understanding these ageist stereotypes: competence and warmth (Fiske et al., 2002). Stereotypes of outgroups are often characterized by differing combinations of these dimensions (Apriceno et al., 2021; Vervaecke and Meisner, 2020). Benevolent ageism reflects perceptions of older people as high in warmth but low in competence and may lead to paternalistic behavioral responses (Vervaecke and Meisner, 2020; Visintin, 2021). Conversely, hostile ageism reflects perceptions of older peoples as both low in warmth and competence and is associated with behaviors involving active harm (Cary et al., 2017; Visintin, 2021). Cary et al. (2017) developed the Ambivalent Ageism Scale (AAS) to measure both hostile and benevolent ageism based on the dimensions of the SCM.

### 1.3 Older Australians in the media during COVID-19

Australian media coverage of the pandemic played an important role in shaping public perceptions of older adults (Reyes-Sosa et al., 2020). Social Representation Theory (SRT) describes how societies construct shared ideas and beliefs that contribute to social order and collective understanding (Cluley and Green, 2019). Media representations are therefore important within this framework, particularly in shaping how older adults were portrayed during the COVID-19 pandemic (Martikainen and Sakki, 2021). A study exploring media portrayals of different age groups during the pandemic in Finland found that older adults were often portrayed as isolated and lonely, whereas younger and middle-aged adults were depicted as experts and caregivers (Martikainen and Sakki, 2021). These portrayals reflected the ‘vulnerability narrative’ of benevolent ageism identified by Cohn-Schwartz and Ayalon (2020), which was also evident in Australian media.

A headline from an Australian news website stated, “Coronavirus in Australia: How to protect elderly, who are most at risk of death”, with the subheading “Protecting the elderly” (Molloy, 2020). Avers et al. (2011) note that ‘elderly’ is an ageist term due to its association with frailty, and thus recommend the use of ‘older adults’ in both media and other contexts. Similarly, an ABC News (2020) article quoted then Australian Prime Minister Scott Morrison as stating, “as a young person you don’t want to be responsible for the severe and possibly fatal disease of an elder, vulnerable Australian”. Such messaging may imply that younger people are relatively unaffected by the virus, while positioning them as responsible for protecting older people, thereby reinforcing paternalistic attitudes and behaviors toward older Australians (Ehni and Wahl, 2020; Whatley et al., 2020).

While these sentiments reflected benevolent ageism, evidence of hostile ageism, or the ‘burden narrative’ was also observed in Australian media reports. Notably, Kehoe (2020) published an article titled “Lives matter but at what cost?”, in which he stated, “Is a person who has lived into their late 70s, 80s or 90s owed the same priority to preserve life as a person in their 20s or 30s who typically has more than 50 years still to live?” Kehoe (2020) also discussed the economic and social costs associated with COVID-19 in ways that positioned older adults as a potential burden on the Australian economy and society. While expressions of hostile ageism became more visible during the pandemic, they may not have been as widely endorsed as expressions of benevolent ageism (Barrett et al., 2020; Swift and Chasteen, 2021).

### 1.4 Social cohesion

Portrayals of older adults during the pandemic raised concerns about increasing intergenerational tension and disruption to social cohesion (Ayalon, 2020; Ehni and Wahl, 2020; Fraser et al., 2020). During times of crisis such as a global pandemic, a shared social identity can strengthen collective resilience (Neville et al., 2021). However, observed intergenerational tensions may diminish this potential for collective resilience (Ayalon, 2020; Visintin, 2021). The pandemic also drew attention to perceived differences between age groups, particularly between older and younger people. Media portrayals of older adults as especially vulnerable to COVID-19 may have encouraged psychological distancing from older people, potentially contributing to ageist stereotyping and discrimination (Meisner, 2020; Swift and Chasteen, 2021). The portrayal of older adults as a vulnerable and homogenous group may therefore weaken social cohesion by exacerbating intergenerational tension (Swift and Chasteen, 2021; Terracciano et al., 2021).

Delhey and Dragolov (2015) found that social cohesion was positively associated with an individual’s subjective wellbeing and optimism about the future. Similarly, Tse et al. (2021) found that, during the COVID-19 pandemic, engagement in prosocial and socially cohesive behaviors was associated with greater psychological wellbeing. Given the relationship between ageism, social cohesion, and wellbeing, it was hypothesized that:

*H*_1_: Greater endorsement of ageist statements would be associated with a greater decline in emotional coping following the onset of the COVID-19 pandemic.

### 1.5 Terror management theory

The endorsement of ageist beliefs may not only disrupt social cohesion at the societal level but also have consequences for individuals. Specifically, the belief that older adults are vulnerable and susceptible to COVID-19 could trigger ageism through the mechanism of death anxiety (O’Connor and McFadden, 2012; Swift et al., 2017). Terror management theory posits that ageism may be explained by the association between older people and death or illness, particularly in a context where mortality is salient (Arcieri, 2021; Greenberg et al., 1986; Swift et al., 2017; Swift and Chasteen, 2021). Consequently, individuals experiencing death anxiety may also fear aging or identifying with older people, thereby reinforcing ageist beliefs (Swift and Chasteen, 2021).

Aging anxiety, particularly among middle-aged adults, has also been associated with increased depression and loneliness (Bergman and Segel-Karpas, 2021; Curșeu et al., 2021; Lamont et al., 2015; Swift et al., 2017). Anxiety about aging may be directly related to negative perceptions of aging processes, including the belief that illness or functional decline are inevitable consequences of aging (Sarkisian et al., 2005). Accordingly, individuals with more negative expectations regarding aging may also be more likely to endorse ageist beliefs. It was therefore hypothesized that:

*H*_2_: More negative expectations regarding aging would be associated with greater endorsement of ageist beliefs.

### 1.6 The contact hypothesis

Allport’s (1954) contact hypothesis asserts that contact between members of different groups can reduce prejudice and increase acceptance of outgroup members. This theory has been supported by a substantial body of evidence, particularly in the context of ageism (Harwood et al., 2005; Helphrey et al., 2019). Researchers have demonstrated that this relationship may be stronger for contact *quality* than for contact *quantity* alone (Bousfield and Hutchison, 2010; Visintin, 2021). It was therefore expected that these associations would also be evident in the context of COVID-19. Accordingly, it was hypothesized that:

*H*_3_: More frequent positive contact with older adults would be associated with lower endorsement of ageist beliefs, less frequent negative contact, and more positive expectations regarding aging.

*H*_4_: More frequent negative contact with older adults would be associated with greater endorsement of ageist beliefs, less frequent positive contact, and more negative expectations regarding aging.

The different theoretical perspectives used in this study help explain ageism at several levels. The Risks of Ageism Model and Stereotype Content Model describe how age-related stereotypes may be internalized and expressed in hostile or benevolent ways, while Social Representation Theory considers how these beliefs are shaped and reinforced through shared social and media representations. Terror management theory may also help explain why heightened attention to illness, aging, and mortality during COVID-19 could increase the salience of age-based stereotypes. In contrast, the contact hypothesis focuses on how interactions with older adults may shape ageist attitudes, while social cohesion considers the broader consequences of age-based divisions during a period of collective stress. Together, these perspectives informed the study’s examination of ageist beliefs, aging expectations, emotional coping, and positive and negative contact with older adults.

### 1.7 The current study

This study aimed to explore ageism, and attitudes toward aging in Australia, during the COVID-19 pandemic. Specifically, it examined associations between perceived changes in emotional coping, ageist beliefs, expectations regarding aging, and the quality of contact with older adults. Despite the growing body of empirical literature, these specific associations have been relatively underexamined within the Australian context. Thus, the current study aimed to contribute to the emerging body of research.

## 2 Materials and methods

### 2.1 Design and participants

The study used an online, cross-sectional correlational survey design to examine associations among perceived changes in emotional coping, expectations regarding aging, hostile ageism, benevolent ageism, and contact quality with older adults.

A total of 273 Australian residents (49 male, 222 female, 2 other) completed the online study. Participants ranged in age from 19 to 82 years (*M* = 40.73, *SD* = 14.74) and were recruited through word of mouth, snowball sampling, and social media. Given the exploratory nature of the study, no *a priori* power analysis was conducted. Sample size was determined with reference to previous survey studies of similar populations and scope. The study criteria required participants to be at least 18 years of age and currently residing in Australia.

### 2.2 Materials

A combination of pre-existing and novel measures was used in this study. An adapted form of the AAS, created by Cary et al. (2017) was used to measure benevolent and hostile ageism among participants. The ERA-12, created by Sarkisian et al. (2005) was used to measure expectations regarding aging. Measures of quality and quantity of contact with older adults were adapted from Walker et al. (2019). A measure asking participants for two adjectives that ‘described older adults’ was included to provide additional insight into participants’ age-related attitudes. Finally, participants reported perceived changes in emotional coping since March 2020 to assess the emotional impact of the pandemic.

Participants were invited to complete the current study via a link to an online survey hosted on the Qualtrics™ survey platform. The study comprised of the Ambivalent Ageism Scale (AAS) adapted for this study, the Expectations Regarding Aging survey (ERA-12), and a measure of contact quality with older adults. The survey also included measures of change in participants’ emotional coping since March 2020. Standard demographic information was also collected, alongside participants’ income bracket, state of residence, occupation, and education level.

#### 2.2.1 Emotional coping

Participants completed a novel two-item measure assessing perceived changes in their ability to cope emotionally with the impacts of COVID-19. The measure was developed for the current study because no suitable previously published measure was identified. Emotional coping was treated as distinct from psychological distress, depression, anxiety, and broader psychological wellbeing, as the measure focused specifically on participants’ own perceptions of how well they were coping emotionally before and after the onset of the pandemic. Responses were recorded on a 5-point Likert scale ranging from 1 (*not well at all*) to 5 (*extremely well*). Participants rated their emotional coping both before and after the onset of COVID-19. For example, participants were asked, “Please select the option that describes how well you were coping emotionally prior to the impacts of COVID-19 (before March 2020)”. A second item assessed coping after March 2020. A change score was calculated by totaling the response for each question, then subtracting the ‘after’ score from the ‘before’ score to produce scores ranging from −4 to 4. Scores below 0 indicated a perceived decrease in coping following March 2020, scores of 0 indicated no change, and scores above 0 indicated a perceived increase in coping.

#### 2.2.2 Expectations regarding aging (ERA-12)

The Expectations Regarding Aging Survey (ERA-12; Sarkisian et al., 2005) was included to assess participants’ expectations regarding the aging process across physical health, mental health, and cognitive functioning. Participants completed 12 items using a 4-point Likert scale ranging from 1 (*definitely true*) to 4 (*definitely false*). Items include statements such as “The human body is like a car: When it gets old, it gets worn out” and “It’s normal to be depressed when you are old”. A total ERA-12 score was calculated using the established algorithm: the summed item score was reduced by 12, multiplied by 25, and divided by 9. Higher scores indicated more positive expectations regarding aging, while lower scored indicated stronger expectations of decline with age (Sarkisian et al., 2005). The scale does not assess knowledge of aging and thus does not include correct or incorrect responses (Whatley and Castel, 2020). The ERA-12 comprises three subscales assessing physical health, mental health, and cognitive functioning. The scale has demonstrated acceptable to good internal consistency in samples of older and middle-aged adults (*α* = 0.70–0.89; Joshi et al., 2010; Sarkisian et al., 2005).

#### 2.2.3 Ambivalent ageism scale (AAS)

The 13-item Ambivalent Ageism Scale developed by Cary et al. (2017) was adapted for the current study to assess hostile and benevolent ageism in the context of COVID-19. Seven items from the original scale were retained: four benevolent ageism items (items 3, 6, 8, and 9), three hostile ageism items (items 10, 11, and 13). One additional hostile ageism item was created for the current study: “Old people have got in the way of me getting vaccinated against COVID-19”, resulting in two four-item subscales. Five distractor items were also presented, including “Australia’s management of COVID-19 has been better than most other countries” to maintain the COVID-19 context and reduce demand characteristics. The measure originally included three subscales: hostile ageism, cognitive weakness, and unwanted help, with the latter two measuring elements of benevolent ageism (Cary et al., 2017). Given the novel nature of COVID-19, there are limited scales assessing attitudes toward older adults specific to this context. Further, it was important to ensure that the context of COVID-19 was made salient during the study. Thus, the AAS was adapted for use in the current research by selecting items with the closest real-world applicability to COVID-19. Responses responded to all items using a 7-point Likert scale ranging from 1 (*Strongly Disagree*) to 7 (*Strongly Agree*). Mean scores were calculated separately for the hostile and benevolent ageism subscales by summing the relevant items and dividing by four. Higher scores indicated stronger endorsement of the corresponding form of ageism. Because the original AAS was substantially modified, psychometric evidence reported for the original scale cannot be assumed to apply to the adapted version used here. Internal consistency for the adapted hostile and benevolent subscales was therefore assessed in the present sample and is reported in the Results.

#### 2.2.4 Qualitative descriptors

Participants were asked to “Please choose two adjectives/words you feel best describe older adults in general” and were provided with two text boxes to enter their responses. The responses were then coded according to whether they reflected hostile or benevolent ageism based on the dimensional categories of the Stereotype Content Model. Two authors (MK, TLS) independently read the pairs of adjectives multiple times to ensure accuracy and assigned the categories, and later met to discuss any uncertainties. Frequencies of individual descriptors were also recorded.

#### 2.2.5 Contact quality

Positive and negative contact with older adults was assessed using two single-item measures adapted from Walker et al. (2019). Participants responded to “On average, how frequently do you have POSITIVE/GOOD contact with older adults?” and “On average, how frequently do you have NEGATIVE/BAD contact with older adults?” using a 7-point Likert scale ranging from 1 (*never*) to 7 (*extremely frequently*). Higher scores indicated more frequent positive or negative contact, respectively. These single-item measures have demonstrated predictive validity and consistent effect sizes across previous studies (Walker et al., 2019).

### 2.3 Procedure

Participants were recruited through advertisements on social media and participated by clicking a link to an online Qualtrics survey. Respondents first viewed the participant information sheet and ethics information, and then indicated their consent to participate before proceeding. Questions then gathered demographic information regarding participants’ age, gender, state of residence, occupation, education level, and household income. Participants then completed the measure of emotional coping. Next, respondents completed the adapted AAS, followed by measures of contact quality with older adults. Participants then reported two words that described older adults, completed the ERA-12, and were provided with a response box to add any additional comments about the study. The presentation order of these scales was chosen specifically to minimize demand characteristics or potential confounding effects associated with presenting the ERA-12 before the adapted AAS. The study took participants approximately 10 min to complete. Participants with missing responses were retained for analyses where the required variables were available.

## 3 Results

### 3.1 Internal reliability and data screening

The ERA-12 scale demonstrated good internal reliability (*α* = 0.86). As the measures of benevolent and hostile ageism were adapted from the original AAS, the internal reliability of each subscale was assessed. The hostile ageism subscale demonstrated acceptable internal reliability (*α* = 0.78), whereas internal reliability was questionable for the benevolent ageism subscale (*α* = 0.68). Sample sizes varied slightly across analyses due to missing responses on individual measures. No imputation was performed, and analyses were conducted using all available data for each relevant measure.

The data were split based on participant age for exploratory analyses, and those who were 65 years of age or older formed the *older adult* group (*N* = 32), and other participants between 18 and 64 years of age formed the *younger adult* group (*N* = 234).

#### 3.1.1 Parametric assumptions

To ensure suitability of variables for parametric analyses, descriptive statistics including normality, linearity, multicollinearity, and homoscedasticity were explored. All variables had an acceptable skewness and kurtosis according to the criteria proposed by George and Mallery (2016), with none deviating beyond the recommended ±2. Several outliers were identified among the data across multiple variables. On closer inspection, most outliers did not appear to represent sampling or measurement errors, and thus were retained for analysis. One outlier was excluded from analysis due to apparent measurement error. Significant deviations from linearity were also observed among several variables. Given these violations, non-parametric correlational analyses were conducted.

### 3.2 Descriptive statistics

Descriptive statistics of the six focal continuous variables were explored and can be found in Table 1.

**Table 1:** Means (M), standard deviations (SD), and ranges of focal continuous variables.

Variable	*M*	*SD*	Range
Emotional Change1 (*not well at all*) to 5 (*extremely well*), pre- to post-COVID	−0.93	1.20	−4 – 3
Financial Change1 (*not well at all*) to 5 (*extremely well*), pre- to post-COVID	−0.43	1.04	−4 – 2
Benevolent Ageism1 (*Strongly Disagree*) to 7 (*Strongly Agree*)	4.07	1.05	1–7
Hostile Ageism1 (*Strongly Disagree*) to 7 (*Strongly Agree*)	2.62	1.16	1–7
ERA-124-point Likert scale ranging from 1 (*definitely true*) to 4 (*definitely false*)	52.16	16.48	8.3–97.2
Positive Contact (*frequency*) 7-point Likert scale ranging from 1 (never) to 7 (*extremely frequently*)	4.94	0.70	2–6
Negative Contact (*frequency*)7-point Likert scale ranging from 1 (*never*) to 7 (*extremely frequently*)	2.88	1.06	1–5

### 3.3 Correlational analyses

A series of Spearman’s rank-order correlations were conducted between the six main continuous variables. Correlational coefficients are presented in Table 2.

**Table 2:** Spearman rank-order correlations of focal continuous variables.

Emotional coping change	(1)	(2)	(3)	(4)	(5)	(6)
(1) Financial Coping Change	–	0.026	−0.03	−0.11	−0.06	−0.03
(2) ERA-12		–	−0.10	−0.22**	0.20**	−0.17**
(3) Benevolent Ageism			–	0.04	0.14*	−0.20**
(4) Hostile Ageism				–	−0.22**	0.32**
(5) Positive Contact					–	−0.35**
(6) Negative Contact						–

*p < 0.05, **p < 0.001.

#### 3.3.1 Expectations regarding aging

No significant association was identified between *changes in emotional coping* and *ERA-12* scores. More positive expectations regarding aging were significantly associated with lower hostile ageism. However, there was no significant association between *ERA-12* scores and *benevolent ageism*.

#### 3.3.2 Positive contact

More frequent positive contact was significantly associated with more positive expectations regarding aging and greater endorsement of benevolent ageism. More frequent positive contact was also associated with lower hostile ageism and less frequent negative contact.

#### 3.3.3 Negative contact

More frequent negative contact was significantly associated with less positive expectations regarding aging and lower benevolent ageism. More frequent negative contact was also associated with greater hostile ageism.

### 3.4 Content analysis of adjectives

A deductive content analysis was conducted using the adjectives participants provided to describe older adults. Participants’ two adjectives were read and then categorized based on whether their choice of adjectives overall represented hostile ageism or benevolent ageism based on the dimensional categories of the Stereotype Content Model (SCM). Separate dichotomous variables were created for hostile and benevolent adjectives, with participants classified as either having or not having used an adjective in each category. For example, if there was evidence of low warmth and low competence across one or both adjectives, the participant was scored as ‘yes’ for *hostile adjectives* and ‘no’ for *benevolent adjectives*. Responses were reviewed multiple times to support consistency in categorization.

Mann–Whitney *U* tests were conducted to compare hostile and benevolent ageism scores between participants who did and did not use corresponding ageist adjectives. The U and z statistics and mean ranks are presented in Table 3. Participants who used hostile ageist adjectives reported significantly higher hostile ageism scores than those who did not. Similarly, participants who used benevolent ageist adjectives reported significantly higher benevolent ageism scores than those who did not.

**Table 3:** Mann–Whitney *U* tests comparing ageism scores according to the use of corresponding ageist adjectives.

Variable	*U*	*Z*	Adjectives – yes		Adjectives – no	
			Mean rank	*N*	Mean rank	*N*
Hostile Ageism	2252.5	−6.68**	201.83	51	120.74	220
Benevolent Ageism	4,517	−2.17*	157.42	51	131.03	220

*p < 0.05, **p < 0.001.

Frequencies of adjectives were recorded and the most common words for each form of ageism are listed in Table 4.

**Table 4:** Frequencies of most common adjectives corresponding with hostile and benevolent ageism that participants used to describe older adults.

Benevolent ageism	Frequency	Hostile ageism	Frequency
Vulnerable	16	Stubborn	15
Slow	8	Entitled	6
Frail	5	Selfish	6
Fragile	4	Ignorant	4

### 3.5 Exploratory age-group analyses

#### 3.5.1 Older and younger adult comparisons

A series of Mann–Whitney *U* tests were conducted to explore differences between older and younger adults’ responses across the six focal variables. Table 5 provides *U* and *Z* values for statistically significant tests, and mean rank values for the significant group differences.

**Table 5:** U-statistics, Z-statistics, N-values, and mean ranks for Mann–Whitney *U* tests assessing differences in expectations regarding aging (ERA-12), positive contact between older and younger adults, emotional coping change, hostile ageism, benevolent ageism.

Variable	*U*	*Z*	Older adults		Younger adults	
			Mean rank	*N*	Mean rank	*N*
ERA	2448.5	−3.07*	170.00	32	126.15	230
Positive Contact	2843.0	−2.46*	159.66	32	128.75	232
Emotional Coping Change	5,535	−5.40**	105.75	108	156.04	163
Hostile Ageism	2252.5-	−6.68**	201.83	51	120.74	220
Benevolent Ageism	4,517	−217*	157.42	51	131.03	220

*p < 0.05.

There were significant differences in *ERA-12* scores between the groups, such that *older adults* had significantly more positive aging expectations than *younger adults*, as depicted in Figure 1. *Older adults* had significantly more positive contact with other older adults than did *younger adults*. All other tests yielded no significant differences between the groups.

**Figure 1:**
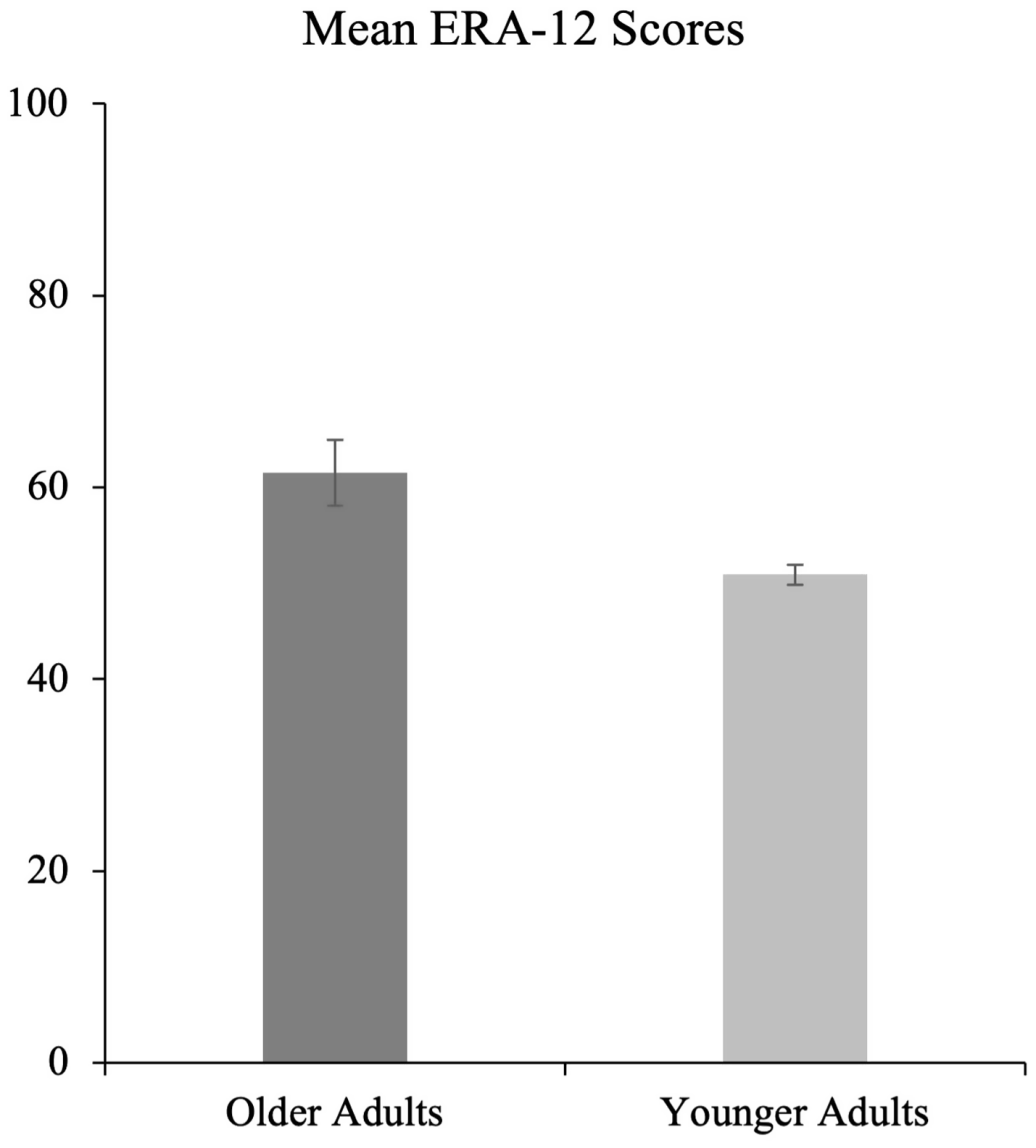
Means and standard errors of expectations regarding aging (ERA-12) scores for older and younger adult participants.

The data were split based on age groups and a series of bivariate correlations tested associations between the six focal variables. A significant inverse relationship between *ERA-12* scores and *hostile ageism* was identified among the *younger adult* group only. This was such that those with more negative aging expectations endorsed more hostile ageist beliefs. Further, significant relationships were identified between *positive contact* and each type of ageism, and *negative contact* with each type of ageism in the same direction as the whole sample. However, these relationships were only identified among younger adults as depicted in Table 6.

**Table 6:** Spearman correlations of variables of note where relationships differed between older adults and younger adults.

Variables		Older adults (*r*_s_)	Younger adults (*r*_s_)
ERA-12	Hostile Ageism	−0.28	−0.21**
Negative Contact	Hostile Ageism	0.24	0.32**
Negative Contact	Benevolent Ageism	−0.09	−0.23**
Positive Contact	Hostile Ageism	0.11	−0.23**
Positive Contact	Benevolent Ageism	0.01	0.18**

**p < 0.001.

## 4 Discussion

This study explored ageism, expectations regarding aging, and the quality of contact with older adults during the COVID-19 pandemic, using an online, cross-sectional correlational study design, and a combination of established and novel measures.

Both benevolent and hostile expressions of ageism were evident during the COVID-19 pandemic, particularly in media representations of older adults (Ayalon, 2020; Meisner, 2020; Whatley et al., 2020). Research in the United States found that those with more negative expectations about aging also reported more unpleasant mood (Whatley et al., 2020). Given the prevalence of ageist messaging during COVID-19 and its potential consequences, it is important to understand the nature and correlates of these beliefs. Examining ageism during a global pandemic may also provide insight into how ageist attitudes operate under conditions of heightened uncertainty, social disruption, and intergenerational tension.

Overall, the findings showed a complex pattern across the study variables. Emotional coping was not associated with ageist beliefs, whereas more positive aging expectations were associated with lower hostile ageism but not benevolent ageism. Associations with intergenerational contact also differed across the two forms of ageism, with positive and negative contact showing contrasting relationships with hostile and benevolent ageism. Exploratory analyses also identified some differences between younger and older adults.

### 4.1 Discussion of findings

#### 4.1.1 Ageism and aging expectation

Contrary to predictions, there was no relationship between perceived changes in emotional coping and endorsement of ageist beliefs. Previous research identified that decreased social cohesion and intergenerational tension were related to poorer mental wellbeing (Delhey and Dragolov, 2015). Accordingly, in the context of COVID-19 where ageism and intergenerational tension were prominent, it was hypothesized that ageism would also be related to lower wellbeing (Swift and Chasteen, 2021; Terracciano et al., 2021). One possible explanation for this finding relates to the measure used to capture emotional wellbeing. While the measure in the present study was useful in assessing changes to emotional coping across time, it likely did not provide sufficient information about an individual’s current level of wellbeing. Consequently, this measure may not have been appropriate for examining this specific association and therefore may explain the lack of relationship found.

Similarly, despite literature suggesting that ageism may be related to social discord, the endorsement of ageist beliefs may not reflect behavioral intentions that would decrease social cohesion, thus explaining these unexpected findings (Swift and Chasteen, 2021). Future research should consider the development of measures to explore coping specifically within the COVID-19 context. Similarly, established measures of social cohesion, or ageist behavioral intentions, should be used to explore their relationships with ageism in the context of COVID-19.

Similarly, more negative ERA-12 scores were associated with greater hostile ageism, but there was no relationship between aging expectations and benevolent ageism. The questionable reliability of the benevolent ageism subscale may have contributed to the lack of relationship found. Moreover, a moderate correlation between the benevolent and hostile ageism subscales was identified by the measure’s creators (Cary et al., 2017). However, the current study found no significant association between the two, highlighting further possible issues with one or both subscales.

As the AAS was adapted for use in the study, its structure differed from the original measure, which may have impacted its psychometric properties (Cary et al., 2017). While this was done to increase its relevance to the study context, it may have resulted in less internal consistency between items. Given the timing of this study, future research should use an improved measure to assess whether these perspectives and attitudes persisted in the aftermath of COVID-19.

#### 4.1.2 Contact quality

The contact findings also differed according to the form of ageism considered. More positive contact with older adults was associated with more positive aging expectations, less negative contact, and lower hostile ageism. However, more positive contact was also associated with greater benevolent ageism. Conversely, more negative contact was associated with greater hostile ageism but lower benevolent ageism. This unexpected finding may be explained by the self-report nature of the measures of contact. Specifically, an individual may complete an unsolicited ‘good deed’ for an older adult and therefore report this as positive contact, while remaining unaware of the harmful consequences of paternalistic beliefs.

These findings may also provide insight into an area of the literature that currently holds mixed support (Visintin, 2021). Specifically, previous research has indicated that intergroup contact, and particularly the quality of that contact, is one of the most reliable mechanisms for reducing prejudice and increasing positive attitudes toward outgroups (Allport, 1954; Bousfield and Hutchison, 2010). Given that intergroup contact may act as a mechanism for reducing homogenous perceptions of a group, it is possible that more positive contact could be associated with less benevolent ageism (Islam and Hewstone, 1993). However, other literature has indicated that positive intergroup contact may reinforce the societal status quo, and thus perpetuate benevolent views of older adults as incompetent (Durrheim et al., 2014; Visintin, 2021). Similarly, recent research found that when healthcare professionals had more contact with older adults, they had more positive attitudes, however also engaged in more benevolent stereotyping (Swift et al., 2017). Indeed, research has explored how ageism differs according to context, and the relevant stereotypes that are salient (Swift et al., 2017). Thus, it is possible that in some contexts, contact may be associated with increased benevolent ageism. Consistent with research in healthcare settings, the current study may represent a relationship between contact and benevolent ageism that exists in a context where negative stereotypes about the competence and health of older adults are salient.

#### 4.1.3 Age-group differences

Exploratory analyses identified differences between older participants (i.e., 65 years and older) and younger participants (i.e., 64 years and younger). First, results indicated that older adults had more positive expectations about aging processes than younger adults. These findings were consistent with previous literature that has consistently demonstrated this relationship and indicated the presence of protective factors against ageism (Chopik and Giasson, 2017; Kornadt et al., 2017). Additionally, these findings corroborate the empirical findings of Whatley et al. (2020) who found that this association was evident in the context of COVID-19. Second, results revealed that older adults had significantly more positive contact with other older adults than did younger adults.

Furthermore, the relationships identified between contact and ageism in the full sample of participants were found to exist only among younger adults. Similarly, negative expectations about aging were associated with more hostile ageism only among younger adults. These results indicated support for the intergroup contact hypothesis given that the effect of contact on prejudice has been demonstrated between members of different groups (Allport, 1954). However, it is important to note the difference in sample sizes between older and younger adults, which may have influenced statistical power and the findings obtained.

Other factors related to sampling and methodology also represented limitations of the current study. First, participants were predominantly from the state of Queensland, Australia and thus may not have provided a representative perspective of all Australians. The use of non-probability recruitment methods may have introduced self-selection bias, and the substantial overrepresentation of female participants further limits the generalizability of the findings to Australian adults more broadly. Finally, the current study was conducted during a specific period in early 2021 and given the variations in the number of COVID-19 cases, lockdowns, preventative health measures, and political and social circumstances throughout the pandemic, results may have differed if the study had been conducted at a later time.

### 4.2 Practical implications

The findings of this study suggest that ageist perceptions of older adults were evident during COVID-19 in Australia. Specifically, perceptions of older adults as ‘vulnerable’ represented a common viewpoint among participants and reflected ageist media sentiments. In addition, over one third of participants used descriptive words that were characteristic of benevolent and hostile ageism when describing older adults. Despite this, endorsement of hostile and benevolent ageist statements was relatively low, although this may be due to the previously described limitations of these measures.

The different relationships observed between contact, hostile ageism, and benevolent ageism have implications for how intergenerational contact is considered in ageism-reduction approaches. While the correlational nature of this research limits interpretations of causality and the direction of relationships, this study makes an important contribution to the literature on ageism during COVID-19 in Australia. Specifically, this study contributes to a growing body of research exploring the nature of benevolent ageism and its relationship with contact.

Furthermore, the present study provided insight into a range of factors related to ageism in the context of a global pandemic. This may support the identification of mechanisms underlying ageism that could be targeted at a societal level to reduce age biases and discrimination. Practically, the findings suggest that interventions involving intergenerational contact may be useful for reducing hostile ageism. However, the association between more positive contact and greater benevolent ageism indicates that contact alone may not be sufficient and that the nature and quality of intergenerational interactions should be considered carefully. This is consistent with evidence that intergenerational contact can be useful in reducing ageism, while also suggesting that different forms of ageism may be affected differently by contact-based approaches (Burnes et al., 2019). Given the detrimental impact of ageism on the health and wellbeing of older adults, it is imperative that research explores factors that can decrease ageism, including more subtle and paternalistic forms. For example, further research could build on the preliminary findings of the current study by exploring how benevolent ageism relates to aging expectations, and how life stressors may impact this relationship.

### 4.3 Conclusions and summary

In conclusion, the present study contributed to a growing body of research on ageism in the context of COVID-19. These findings extended existing research on the relationships between contact quality and ageism, providing insight into the perceptions of a mixed-age sample of Australians toward older adults. Further, this research contributes to the understanding of issues during a global pandemic that should be addressed to meet the needs of Australia’s aging population.

While this study focused on ageism and its impacts in the context of COVID-19, ageism is a longstanding societal issue. Thus, it is vital that research builds on findings from studies conducted during the pandemic to inform strategies for strengthening intergenerational cohesion and reducing ageism. As most individuals expect to 1 day become older adults, ageism is an issue that affects everyone.

## Data Availability

The raw data supporting the conclusions of this article will be made available by the authors, without undue reservation.
